# Editorial: On-Orbit Servicing and Active Debris Removal: Enabling a Paradigm Shift in Spaceflight

**DOI:** 10.3389/frobt.2019.00136

**Published:** 2019-12-12

**Authors:** Markus Wilde, Jan Harder, Enrico Stoll

**Affiliations:** ^1^ORION Laboratory, Department of Aerospace, Physics and Space Sciences, Florida Institute of Technology, Melbourne, FL, United States; ^2^Munich School of Robotics and Machine Intelligence, Technische Universität München, Munich, Germany; ^3^Enrico Stoll, Institute of Space Systems, Technische Universität Braunschweig, Brunswick, Germany

**Keywords:** on-orbit servicing (OOS), space debris removal, space robotics and automation, guidance, navigation and control (GNC), rendezvous and capture

As of April 2019, the satellite database maintained by the Union of Concerned Scientists showed 2,062 active satellites orbiting the Earth (Union of Concerned Scientists, 2019). They provide services essential to global security, commerce, science, and the safety and well being of large parts of the population. Some examples are global communications and navigation, remote sensing, climate science, and weather observation. Although many global systems such as military communications, commercial logistics, or weather forecasting can no longer function without space based services, spacecraft manufacturers, owners, and operators are surprisingly bad shepherds of the orbital environment. For most of the history of spaceflight, satellites have been treated as disposable articles and the orbits around Earth as an infinite resource. With about 5,400 space missions flown since 1957, NORAD tracks almost 20,000 objects in orbit around Earth, of which more than 12,100 are classified as debris objects and another 2,200 are spent upper stages. This is before the “mega constellations” proposed by a number of new commercial space enterprises add a projected 20,000 new satellites (Henry, 2018, 2019). If the historical rate of 9% of all satellites experiencing a major malfunction before their end of life persists, spaceflight may become unsustainable (Long et al., 2007).

On-orbit servicing and active debris removal can be part of the solution. If satellites can be inspected and repaired in orbit, potentially crippling malfunctions can be identified and mitigated without the satellite becoming part of the debris population. If a satellite still becomes inoperable, it can be safely removed from orbit. If healthy satellites can be refueled in orbit, they can be operated beyond their original design life, even given the necessity of collision avoidance maneuvers or orbital changes to optimize ground coverage. Furthermore, if subsystems and components of satellites are replaced throughout their lifetimes, spacecraft can be continuously upgraded to match market demand and to avoid technical and economic obsolescence. Therefore, operational on-orbit servicing systems can increase spacecraft capability and longevity, can provide flexibility in design and operations, and hence increase the overall return on investment on space systems.

The value of on-orbit servicing was clearly demonstrated by the servicing missions executed by the Space Shuttle, most notably to the Hubble Space Telescope (Goodman, 2006; Joppin and Hastings, 2006). These missions clearly showed how on-orbit servicing can save a space mission and then continuously extend the lifetime and improve capabilities. Due to the high cost and risk associated with human spaceflight, the emphasis was soon placed on developing robotic systems able of replicating the servicing abilities of the Shuttle orbiter/astronaut teaming. This resulted in a number of demonstrator missions for rendezvous, formation flight, capture and servicing technologies, notably Engineering Test Satellite (ETS) VII in 1997 and Orbital Express in 2007 (Yoshida, 2003; Kennedy, 2008). After a brief hiatus, development of on-orbit servicing and debris removal systems is being pursued with renewed vigor, with NASA planning to fly its Restore-L refueling and relocation mission to a client in Low Earth Orbit in 2022 and DARPA continuing with its Robotic Servicing of Geostationary Satellites (RSGS) program (Reed et al., 2016; Roesler et al., 2017).

Before routine robotic servicing and removal missions become a reality, a number of economic, regulatory and technical problems remain to be solved. The primary technical challenges arise from the non-cooperative nature of client satellites and removal targets. With only a few exceptions, no satellite is equipped with sensor fiducials or capture interfaces that would facilitate rendezvous, final approach and capture. Furthermore, a debris object or a satellite in safe mode after a malfunction will not be in a stable attitude but will be tumbling, which makes the difficult task of characterizing its motion, identifying suitable capture features, approaching it, grasping it, stabilizing it and finally executing servicing operations a daunting challenge. Therefore, on-orbit servicing and active debris removal require substantial advances in the fields of sensing, relative navigation, path planning, controls, modeling of dynamic systems, robotics, communications and operations (Moosavian and Papadopoulos, 2007; Fong et al., 2013; Flores-Abbad et al., 2014; Shan et al., 2016; Wilde et al., 2019).

Those are the broad themes of the articles collected in the Research Topic “On-Orbit Servicing and Active Debris Removal: Enabling a Paradigm Shift in Spaceflight.” Sternberg and Miller investigate a parametrized trajectory determination method for the fuel-optimal approach of tumbling targets. Virgili-Llop and Romano and Nagaoka et al. present methods to capture and detumble or despin resident space objects using robotic manipulators. How the complex dynamic interactions between a manipulator and a spacecraft can be modeled and simulated for system design and mission planning purposes is detailed in a tutorial by Wilde et al. The existing types of mechanical, thermal, data, and electrical power connectors available for connecting a robot arm or a servicer spacecraft to a resident space object are reviewed by Yan et al. to lay the foundation for future system developments. Jaekel et al. close the loop between fundamental research and actual systems design and operations planning by reporting on the robotics development efforts for the ESA e.Deorbit debris removal mission.

The articles presented provide an overview of the current research and development dedicated to taking on-orbit servicing and active debris removal from the stage of laboratory experiments and limited demonstration missions to an operational orbital servicing infrastructure. Having servicing robots routinely roam Earth orbit, removing debris objects and repairing, refueling, maneuvering and upgrading satellites can be a significant stepping stone to a sustainable use of space, and to continued growth of the space economy in Earth orbit, cis-lunar space, and beyond.

## Author Contributions

MW wrote the editorial. All authors contributed to the editorial in equal parts.

### Conflict of Interest

The authors declare that the research was conducted in the absence of any commercial or financial relationships that could be construed as a potential conflict of interest.
